# Tear fluid biomarkers in ocular and systemic disease: potential use for predictive, preventive and personalised medicine

**DOI:** 10.1186/s13167-016-0065-3

**Published:** 2016-07-13

**Authors:** Suzanne Hagan, Eilidh Martin, Amalia Enríquez-de-Salamanca

**Affiliations:** 1 Department of Life Sciences, Vision Sciences, Glasgow Caledonian University (GCU ), G4 0BA Glasgow, Scotland, UK; 2 Institute of Applied Ophthalmobiology (IOBA), University of Valladolid, Valladolid, Spain; 3 Biomedical Research Networking Centre in Bioengineering, Biomaterials and Nanomedicine (CIBER-BBN), Valladolid, Spain

**Keywords:** Biomarkers, Tear fluid, Proteome, Metabolome, Predictive preventive personalised medicine (PPPM), Dry eye, Glaucoma, Diabetic retinopathy, Cancer, Neurological disorders

## Abstract

In the field of predictive, preventive and personalised medicine, researchers are keen to identify novel and reliable ways to predict and diagnose disease, as well as to monitor patient response to therapeutic agents. In the last decade alone, the sensitivity of profiling technologies has undergone huge improvements in detection sensitivity, thus allowing quantification of minute samples, for example body fluids that were previously difficult to assay. As a consequence, there has been a huge increase in tear fluid investigation, predominantly in the field of ocular surface disease. As tears are a more accessible and less complex body fluid (than serum or plasma) and sampling is much less invasive, research is starting to focus on how disease processes affect the proteomic, lipidomic and metabolomic composition of the tear film. By determining compositional changes to tear profiles, crucial pathways in disease progression may be identified, allowing for more predictive and personalised therapy of the individual.

This article will provide an overview of the various putative tear fluid biomarkers that have been identified to date, ranging from ocular surface disease and retinopathies to cancer and multiple sclerosis. Putative tear fluid biomarkers of ocular disorders, as well as the more recent field of systemic disease biomarkers, will be shown.

## Background

Recent developments in the accessibility and sensitivity of proteomic assays have led to the examination of tear fluids as a potential tissue source for biomarker analysis. Tear sampling potentially provides a convenient, non-invasive method of analysing an accessible body fluid for the investigation of biomarkers in predictive, preventive and personalised medicine (PPPM). This review gives an up-to-date overview of how tear fluid analysis is being undertaken to identify novel markers of both ocular and systemic disease.

## Tear fluid analysis in ocular disease

Tears are a complex mixture of proteins, lipids, mucins, water and salts, and a recent study has identified 1526 proteins via proteomics [1], making them less complex (as a body fluid) than serum or plasma. Due to this less complex nature, and also because at the ocular surface the tears represent the “proximal fluid”, the final output of the lacrimal functional unit (LFU [2]), the study of their composition has been proposed as an ideal source for discovering biomarkers associated with the various components of the LFU [3], and there has been increased interest in determining novel tear biomarkers of ocular diseases, e.g. dry eye disease (DED), vernal conjunctivitis, diabetic retinopathy, Graves’ ophthalmopathy, ocular tumours and glaucoma, to name a few [1, 4–8]. Moreover, tears are being investigated for the identification of biomarkers of systemic disease.

A biomarker, as defined by the National Institute of Health (NIH), is

A characteristic that is objectively measured and evaluated as an indicator of normal biological processes, pathogenic processes, or pharmacologic responses to therapeutic intervention [9].

While the US Food and Drug Administration (FDA) describes a biomarker as

Any measurable diagnostic indicator that is used to assess the risk or presence of disease [10].

In the field of PPPM, biomarkers are playing an increasingly important role in the discovery and development of new drugs and point-of-care devices design, as diagnostic tools or for objective monitoring of treatment in clinical trials. As described above, a specific disease biomarker is defined as a measurable characteristic in a biological system which changes due to disease, exposure to chemicals or other factors. Both proteomic and genomic studies are applied to the search for novel and specific biomarkers of disease processes. Biomarkers are key indicators that can provide vital information detecting risk of disease, disease progression, disease activity, prediction of response to therapies or adverse events and drug interactions or establishing baseline risk. A useful biomarker has to correlate with clinical parameters, such as specific symptoms, clinical signs and validated diagnostic tests. Also, whenever possible, non-invasive samples should be used.

Therefore, the utility of biomarkers in personalised medicine for both ocular and systemic disease is very pertinent to the current interest in tear fluid proteomics. This review will seek to provide a timely account of the ongoing global search to identify relevant tear fluid biomarkers, including the proteins of interest and the technologies employed.

### Tear fluid analysis of diseases affecting the ocular surface

#### Dry eye disease

Dry eye disease (DED) is a multifactorial inflammatory disorder of the LFU that is characterised by ocular discomfort, visual disturbances, tear film instability, increased tear osmolarity and inflammation [2, 11]. Research into tear biomarkers has been increasing in DED, mainly due to the fact that in this multifactorial syndrome there is a lack of concordance between clinical symptoms and signs [12–15]. As a consequence, DED diagnosis has been difficult and the development of new pharmacological therapies is hampered by the lack of objective tests for response outcomes in clinical trials [16–18]. For these reasons, there is a growing interest in finding objective biomarkers that could be used as diagnostic tools for DED, or for objective monitoring of treatment in clinical trials.

A large number of tear fluid studies in DED have therefore been performed in the last 5 years [19–22]. These studies comprise research into the different forms of DED, including mild-moderate DED, evaporative cases (including meibomian gland dysfunction (MGD)) or more severe hyposecretor forms of DED, e.g. Sjögren’s syndrome (SS) and ocular graft versus host disease (GVHD). As inflammation is a key component of DED, numerous tear biomarker studies for this disease have included the analysis of inflammatory molecules, such as cytokines/chemokines, as well as other molecules, e.g. growth factors, mucins, neuromediators and lipids. Many of these studies have shown differences in several tear molecules in DED patients compared to healthy subjects, or among the different types of DED. A number of reviews on the use of tears as a source of biomarkers have been published to date, including for non-ocular diseases [23–25] and in reviews specifically dedicated to DED [26–29].

##### Current tear proteins under investigation as DED biomarkers

Various groups have performed tear proteomics analysis in order to determine which proteins and/or protein profiles are specifically related to DED. For example, a proteomic approach to detect tear fluid DED biomarkers was used by Aluru et al. [4] by means of 2D electrophoresis and differential gel electrophoresis (DIGE). The authors found lysozyme proline-rich protein 4 (LPRR4) to be significantly down-regulated in several types of DED. Based on its differential expression and the correlation of LPRR4 tear levels with disease severity, this protein has thus been proposed as a potential biomarker of DED [4]. Other proteomic studies have also reported protein profiles that are specifically related to DED [30–35]. For example, in their study, Grus et al. [30] defined a seven-peptide panel for DED. This panel included calgranulin A/100A8, which was found to be increased in DED patient tears, as well as LPRR3 and LPRR4, nasopharyngeal carcinoma-associated PRP 4 and α-1 antitrypsin. The latter four proteins were found to be decreased in DED [30]. This panel had a 90 % sensitivity and specificity when used together with an artificial neural network.

By using iTRAQ quantitative proteomics, coupled with 2D nano-LC-nano-ESI-MS/MS alongside a statistical model, Zhou et al. [31] identified six up-regulated proteins in tears from DED patients versus controls. These included α-enolase, α-1 acid glycoprotein 1, S100 A8/calgranulin A, S100 A9/calgranulin B, S100 A4 and S100 A11 (calgizzarin), as well as four down-regulated proteins, including prolactin-inducible protein (PIP), lipocalin-1 (LCN-1), lactoferrin and lysozyme [31]. With a four-protein biomarker panel (including α-enolase, PIP, LCN-1 and S100 A9/calgranulin B), this group obtained a diagnostic accuracy for DED of a 96 % (91 % sensitivity and 90 % specificity). Moreover, using iTRAQ technology Tong et al. [32] found that S100 A8/calgranulin A and S100 A9/calgranulin B correlated to MGD severity and redness and that LCN-1 correlated with tearing in non-SS-DED. Several of these tear proteins have been described as being involved in ocular surface defence, with many of them (such as lysozyme, lactoferrin, LCN-1 and defensins) being essential components of the innate immune defence system. In fact, due to the fact that many studies have shown that DED patients present decreased levels, the measure of tear lysozyme and lactoferrin was one of the first proposals of objective tests for DED diagnosis [36–38].

In 2013, Boehm et al. [33] analysed tear protein patterns of dry eye patients by including different clinical phenotypes, e.g. aqueous-deficient dry eye, (DRYaq), lipid-deficient dry eye (DRYlip) and a combination of the two (DRYaqlip), in order to examine their influence on tear film protein composition. By means of a surface-enhanced laser desorption/ionisation-time-of-flight (SELDI-TOF)/matrix-assisted laser desorption/ionisation-time-of-flight (MALDI-TOF)/TOF mass spectrometry (MS)-based strategy to detect candidate biomarkers, followed by a discovery study (study 1) and a validation study (study 2), their results showed that tear LPRR4 was diminished in both DRYaq and DRYaqlip patients, compared to healthy subjects. Moreover, mammaglobin B, lipophilin A, S100A8/calgranulin and beta-2 microglobulin (B2M) precursor were increased in this subgroup. DRYlip patients revealed only slight tear protein alterations and strongly deviated from the DRYaq or DRYaqlip group [33]. By using this six-protein biomarker set, they achieved a DED patient versus healthy subjects discrimination sensitivity and specificity of almost 100 % for the DRYaq and DRYaqlip patients (AUC value = 1).

Based on tear proteome and protein network analyses for tear film characterisation in DED and MGD, Soria et al. [34] have presented a pentamarker panel, including S100A6, annexin A1, annexin A11, cystatin S (CST4) and phospholipase A2-activating protein (PLAA), with an area under the ROC curve of ≥97.7 % (sensitivity ≥94.3 %; specificity ≥97.6 %) for DED versus controls. This panel also discriminated between the DED, MGD and control individuals, with a global correct assignment of 73.2 % between all groups. Versura et al. [35] have also proposed another tear protein panel for its use for DED diagnosis, based on the simultaneous measurement of tear transferrin, LCN-1 and total protein; this panel has a sensitivity of 96 % and a specificity of 98 %.

Proteomic studies in tears from dry eye patients, specifically SS, have also been done. By using SELDI-TOF-MS, Tomosugi et al. [39] found ten protein peaks that could be used to discriminate SS patients from non-SS-DED patients and controls. However, they did not identify the particular proteins. Li et al. [40] also examined the tear film proteome of SS-DED patients compared to non-SS patients with DED symptoms and to normal healthy controls. A total of 435 proteins were identified by 2D nano-LC-MS/MS, and among them, 56, including defensin-1, clusterin and lactotransferrin, were found to be unique to SS-DED patients [40]. Cathepsin S activity measurement in tears has also been proposed as a candidate biomarker for SS [41], as tear activity of this protein was shown to be 4.1-fold higher in SS patients than that in patients with other autoimmune diseases, 2.1-fold higher than that in patients with nonspecific DED and 41.1-fold higher than that in healthy subjects [41]. Anti-SS-A and anti-SS-B, as well as anti α-fodrin antibodies, have also been determined in tear fluids of patients with SS [42–44].

Other types of proteins have also been measured in tears of DED patients. For instance, mucin (MUC)5AC protein tear levels have been shown to be significantly reduced in tears of patients with SS [45]. Guo et al. [21] have measured tear malate dehydrogenase (MDH) 2 in mild DED patients. They found that MDH2 activities in the DED group were significantly increased compared to that in tears from a control (healthy) group. This group also found a significant negative correlation of tear MDH2 with clinical parameters, such as tear production and tear quality values, and a positive correlation with soreness symptoms. Recently, a novel protein of the tear film belonging to the family of surfactant proteins, the palate lung nasal clone (PLUNC), has been found to be increased in DED patient tears [46].

Neuromediators, such as substance P, calcitonin gene-related peptide (CGRP), neuropeptide Y (NPY), vasointestinal peptide (VIP) and nerve growth factor (NGF), have also been determined in tears from DED patients and correlated with clinical findings [47]. Specifically, NGF tear levels were found to be significantly increased in DED patients, whereas CGRP and NPY were significantly decreased, compared to healthy subjects. Tear NGF levels correlated directly, and CGRP and NPY inversely, with DED severity. Also, in a recent study by Chhadva et al. [48], serotonin tear concentration has been correlated to facets of DED and has been found to be significantly higher in those DED patients that presented with both DED symptoms and aqueous tear deficiency, compared to those patients with DED symptoms but normal tear production and those without DED symptoms.

##### Tear cytokines and chemokines in DED

As mentioned above, DED is associated with immune and inflammatory processes, so there are a great number of studies particularly focused on the identification in tears of profiles of cytokines and chemokines in the different clinical subgroups of patients with DED. The development of multiplex assay technologies, such as cytometric bead array (CBA), or XMAP technology, developed by the Luminex Corporation (reviewed in [49]), has made it possible to measure multiple molecules in minute volumes of samples, which has been useful when measuring cytokines in tear samples. Prior to this technological development, tear protein analysis was limited by the sample amount requirement of other analysis techniques.

Several inflammatory cytokines/chemokines (such as IL-1, IL-6, TNF-α, metalloproteinase (MMP)-9, IL-17A, IL-1RA, IL-8/CXCL8, IL-22, INF-γ, MIG/CXCL9, IP-10/CXCL10, I-TAC/CXCL11, macrophage inflammatory protein-1 alpha (MIP-1α/CCL3), MIP-1β/CCL4 and RANTES/CCL5, among others) have been found to be significantly increased in tears from DED patients [19, 22, 50–63]. Whereas endothelial growth factor (EGF) has been found to be significantly decreased [51, 55, 64] in the more severe forms of DED. Other studies have also shown differences in cytokine profiles among the different types of DED forms, e.g. non-SS versus SS-DED, and/or its severity. For example, Boehm et al. [60] found that while the cytokine profile in patients with aqueous-deficient dry eye and dry eye patients with a combination of aqueous-deficient and changes in their lipid layer were quite similar, the cytokine profile of DED patients with only changes of the lipid layer was similar to that of the healthy controls. Furthermore, Tan et al. [62] found that IL-17 and IL-22 were significantly increased, not only in tears from patients with DED (compared to healthy controls), but also in SS patients, compared to non-SS-DED patients. Another study [65] revealed that, besides IL-17A, tears from SS-DED patients presented with increased levels of IL-6, IL-10, IL-4, INF-γ and TNF-α, compared to non-SS-DED and to controls. In another study, Lim et al. [66] analysed IL-21 tear concentrations in primary SS patients and found that its level was significantly increased compared to healthy controls and that it also correlated significantly with ocular surface stain score and tear production (Schirmer’s I test) values in SS patients. Moreover, correlations of inflammatory tear molecule levels such as IL-6, IL-8/CXCL8, TNF-α, IL-1Ra and I-TAC/CXCL11 (among others) with clinical parameters and/or disease severity have been shown in some of those studies, further corroborating the utility of tear analysis for this disease [19, 20, 55, 59–61, 66–68]. Specifically, MMP-9 measurement in tears has already been proposed as a sensitive method for DED severity determination [56, 63], and a commercial point-of-care device has already been developed (InflammaDry®, RPD, USA [69]).

Tear cytokine and chemokine measurement has also been performed in DED patients exposed to different controlled environmental conditions within an environmental chamber. For example, the studies carried out by Dr. Calonge’s group [70–72] have shown that IL-6 and MMP-9 tear levels increase in DED patients, while EGF decreases after exposure to a controlled environment, simulating an in-flight airplane cabin (23 °C, 5 % relative humidity, localised air flow and 750 mb of barometric pressure [70]. This research group has also reported [71] that after a 2-h exposure to a controlled desiccating environment (5 % relative humidity), control non-symptomatic subjects had decreased EGF and increased IL-6 tear levels. Moreover, MMP-9 tear levels were also increased in both DED patients and non-symptomatic controls. Besides, under similar desiccating conditions, DED-SS patients showed not only IL-6 and MMP-9 tear cytokine changes but also IL-1RA and IL-8/CXCL8 tear increased levels [72].

##### Tear lipidome in DED

Besides proteomic studies, the tear fluid lipidome in DED patients has been evaluated, in an attempt to determine the composition and nature of tear lipids (secreted by the meibomian gland) and to identify any alterations in those patients. Differences in the meibomian fatty acid composition in patients with MGD and aqueous-deficient DED were shown by Joffre et al. [73]. Moreover, studies by Lam et al. [74, 75] addressed the meibum lipid composition in DED patients and found several lipid species that were significantly increased in this demographic, including sphingomyelin and phosphatidyl species. They reported significant differences in the tear levels of *O*-acyl-w-hydroxy-fatty acid (OAHFA) species, depending on DED severity. In MGD patients undergoing eyelid-warming treatment, Lam et al. [75] found a reduction in tear fluid lysophospholipid and polyunsaturated fatty acid (PUFA)-containing diacylglyceride species, as well as an increase in some PUFA-containing phospholipids and OAHFAs, upon treatment. This group suggested that the lipidome changes were related to reduced rates of ocular evaporation and an improvement in ocular symptoms of patients [75].

Tears have also been used for the evaluation of lipid oxidative stress status in SS patients. In a study by Wakamatsu et al. [76], tear concentrations of hexanoyl-lysine (HEL) in SS patients were found to correlate significantly with ocular surface staining scores and inflammatory cell density. More recently, Choi et al. [77] evaluated the tear concentrations of HEL, 4-hydroxy-2-nonenal (HNE) and malondialdehyde (MDA) in patients with non-SS-DED and 33 control subjects and found that their levels significantly correlated with clinical tests for ocular surface health, e.g. TBUT, Schirmer’s test score, tear clearance rate, keratoepitheliopathy score, conjunctival goblet cell density and symptom score.

##### Tear metabolome in DED

As shown earlier, numerous proteins and lipids have been identified by means of proteomics and lipidomics of tears in DED. By contrast, the number of identified metabolites is more limited and there are few studies regarding analysis of tear metabolites in DED. In a recent study by Galbis-Estrada et al. [78], the metabolomic profile of reflex tears from DED patients was analysed by nuclear magnetic resonance (NMR) spectroscopy of hydrogen-1 nuclei; their results showed that, when compared to tears from healthy subjects, there were differences in tear composition of cholesterol, *N*-acetylglucosamine, glutamate, creatine, amino-n-butyrate, choline, acetylcholine, arginine, phosphoethanolamine, glucose and phenylalanine levels. In another study from this group [79], they further studied the metabolomic profile of basal tears from DED patients and compared to healthy subjects, both before and after oral nutraceutic supplementation (containing antioxidants and essential PUFAs) after 3 months. Their results showed that there were significant differences in the tear metabolic profile of both groups under study, both pre- and post- supplementation.

##### Tear fluid biomarkers of ocular GVHD-associated DED

Besides SS, another subtype of severe DED is that present in patients that suffer from ocular GVHD. GVHD is an immune-mediated inflammatory disease that haematological stem cell-transplanted (HSCT) patients may develop, in which host tissues are attacked by immunocompetent cells from the donor [80]. Up to 60–90 % of patients have ocular involvement; in particular, chronic GVHD patients develop a very severe form of dry eye. Signs and symptoms of ocular involvement from chronic GVHD may mimic typical DED, but in GVHD patients can lead to a serious abnormality of the ocular surface, affecting patient’s quality of life and eventually leading to permanent visual loss. GVHD-DED is mainly due to aqueous tear deficiency, Sjögren-like, and histology shows inflammatory destruction of the conjunctiva and lacrimal gland with fibrosis, resulting in tear production deficiency [81].

Some studies have already shown that some cytokines have significant different levels in tears of those ocular GVHD patients when compared to healthy subjects or to GVHD patients without ocular involvement. One of those studies, Riemens et al. [82], demonstrated that IL-6 and IFN-γ were significantly increased in tears from ocular GVHD patients. While Sakimoto et al. [83] also described that soluble TNF receptor 1 (sTNFR1) expression was significantly increased in tears from GVHD patients. Recently, Jung et al. [84] also conducted a study in which they studied tear concentration of an 8-cytokine panel in chronic GVHD patients and compared it to HSCT patients that did not develop GVHD. Their results showed that IL-2, IL-10, IL-17A, INF-γ, IL-6 and TNF-α were elevated in patients with GVHD compared to transplanted patients without GVHD. Additionally, they found that IL-10, IL-17A, IL-6 and TNF-α had significant diagnostic abilities, as based on their calculated odds ratio and AUC values. This group also showed that those molecules, along with INF-γ and IL-2, presented significant correlation with clinical parameters, particularly with severity [84]. Cocho et al. [85] have also used tear molecule levels to develop a predictive model, based on a panel of tear cytokines in chronic ocular GVHD. They found that, compared to healthy subjects, these patients had significantly decreased tear levels of EGF and IP-10/CXCL10 and increased levels of IL-1RA, IL-8/CXCL8 and IL-10. Significant correlations with clinical features (including tear production and stability and symptoms, hyperaemia and ocular surface integrity) were also found for these molecules. A statistically generated IL-8/CXCL8 and IP-10/CXCL10 tear level-based predictive model was found to have an AUC value of 0.9004, a sensitivity of 86.36 % and a specificity of 95.24 % [85].

#### Ocular allergies

Tear molecule analysis has also been addressed in cases of ocular allergy. Several clinical subtypes, such allergic conjunctivitis, giant papillary conjunctivitis (GPC, although this remains controversial), vernal keratoconjunctivitis (VKC) and atopic keratoconjunctivitis (AKC), are considered allergy-related disorders [86]. As for the case of DED, tear analysis in ocular allergy patients has revealed significant correlations of several molecules with clinical symptoms and signs, with specific molecule profiles associated to the different clinical subtypes of allergy. Some of these studies have already been reviewed [26, 27, 87]. Many of the tear molecules analysed in ocular allergy are related to cytokines/chemokines, as these molecules have been shown to play a key role in allergy. Although, as in the case of DED, there are also studies of some other proteins and neuromediators.

##### Tear cytokines and chemokines in ocular allergy

Regarding tear cytokine/chemokine analysis in the different subtypes of ocular allergy, one of the first studies addressing this was that of Uchio et al. [88]. Using ELISA assays, this group analysed tear levels of Th-1-IFN-γ and IL-2 and Th-2-IL-4 and IL-5 in VKC, AKC, allergic conjunctivitis (AC) and normal subjects. They found that tear IL-4 levels in AKC patients were significantly higher than those in VKC, AC and controls and that IL-4 tear levels differed significantly in patients with AKC with proliferative lesions versus VKC patients. Also, tear IL-5 levels in patients with diseases associated with proliferative lesions were found to be higher than those in AC and normal controls. Also in this year, Leonardi et al. [89] determined soluble leukocyte activation markers (including neutrophil myeloperoxidase, eosinophil cationic protein (ECP), eosinophil neurotoxin and soluble IL-2 receptor) and histamine, in tears from VKC, AKC, seasonal AC and GPC. Later, this group analysed tear eotaxin-1/CCL11 and eotaxin-2/CCL24 concentrations in VKC and AKC patients (and normal subjects) and found that eotaxin-2/CCL24 was significantly increased in tears from allergic patients [90]. Tear levels of both molecules correlated with the percentage of eosinophils in the tear fluid. Another study by this group showed significantly increased MMP-1 and MMP-9 in VKC patients, compared to controls. Additionally, MMP-9 activity was found to be correlated with corneal involvement and giant papillae formation [91]. MMP-9 tear levels have also been found to be increased in AC patients in a study by Acera et al. [54].

Taking advantage of the development of multiplex cytokine analysis (CBA), Cook et al. [92] analysed tear concentrations of IFN-γ, TNF-α, IL-2, IL-4, IL-5 and IL-10, in both allergic and non-allergic patients. Their study showed that tears from allergic patients presented with decreased levels of IL-10 and also significant increased ratios of TNF-α/INF-γ, IL-5/INF-γ and IL-5/IL-10, versus non-allergic subjects. This was one of the first studies that established the usefulness of the CBA technique for cytokine tear analysis. Also by the CBA method, research by Nivenius et al. [93] analysed the concentrations of IFN-γ, TNF-α, IL-2, IL-4, IL-5 and IL-10 in tears from AKC patients and found that they presented significantly increased tear levels of these molecules. While Leonardi et al. [94] investigated and compared cytokine tear levels among different types of allergic subtypes, including VKC, AKC, chronic and seasonal AC (SAC), they found specific profiles of tear molecules that were significantly increased in each one; IL-1β, IL-2, IL-5, IL-6, IL-12, IL-13 and MCP-1/CCL2 tear levels were found to be increased in all allergic patients groups (compared to controls); IL-4, IFN-γ and IL-10 were elevated in SAC and VKC, while eotaxin-1/CCL11 and TNF-α were only increased in VKC patients group. They also noted significant differences in the expression of IL-5, RANTES/CCL5 and eotaxin-1/CCL11 in VKC patients, compared to that in those with SAC.

By means of a 40-molecule array, Shoji et al. [95] demonstrated that different cytokines were differentially increased or decreased in tears (compared to controls), depending on whether they were from VKC or GPC patients. Particularly, in VKC patients, eotaxin-1/CCL11, IL-11, MCP-1/CCL2 and M-CSF increased to four times the values in the control group, and eotaxin-2/CCL24, IL-4, IL-6, IL-6sR, IL-7, MIP-1δ and TIMP-2 tear levels were increased to eight times the control values. The increase in tear IL-6sR was statistically significant in both the VKC and GPC patients compared to that in the controls, while that in eotaxin-2/CCL24 and that in TIMP-2 were significant only in the VKC group and only in the GPC group, respectively, compared with that in the controls. Whereas in tears from the GPC patients, IL-6, M-CSF and MIG/CXCL9 increased to four times than those in the control group, and eotaxin-2/CCL24, IL-6sR, IL-11, MIP-1δ and TIMP-2 increased to eight times the control values. These same authors also confirmed in another study [96] that in these two groups, tear sIL-6R levels were significantly increased and correlated to clinical score of allergic inflammation of the ocular surface in VKC patients and proposed the use of tear sIL-6R as useful biomarker for patients with AC disease. Another multiplex methodology used for the study of tear molecule concentration is the use of membrane arrays, a stationary phase protein analysis technique. With an optimised protocol, Sack et al. [97] used this method to study 16 inflammatory mediators (GM-CSF, IL-1α, IL-1β, IL-2, IL-3, IL-4, IL-5, IL-6, IL-7, IL-8/CXCL8, IL-10, IL-12, IL-13, INF-γ, MCP-1/CCL2 and TNF-α) in tears from allergic patients, in both open- and closed-eye environments and showed detectable levels of most of the protein panel in these patients, particularly the found enhanced IL-2, IL-4, IL-5 and IFN-γ signals in open eye samples and IL1-α and TNF-α in closed-eye patients’ tear samples, versus controls, in which only IL-8 was detectable.

##### Other tear biomarkers of ocular allergies

Studies have been undertaken on tear molecules from ocular allergy patients, such as histaminase, ECP and histamine. In 1995, these molecules were evaluated by ELISA and RIA in VKC patients [98]. While Montan et al. [99] analysed tear ECP levels of various groups of AC patients (VKC, AKC, SAC and GPC) versus healthy subjects, they found that subjects with AKC and VKC had significantly higher tear ECP values than subjects with GPC and SAC. Additionally, they found that there was a significant correlation between ECP values and disease severity in all disorders [99].

Other studies have addressed the tear analysis of some other molecules in allergic tears such as haemopexin, neuromediators and allergen-specific IgE antibodies [8, 100–103]. Haemopexin tear levels were found to be increased in VKC patients and significantly associated with disease severity [101]. In agreement with those results by Pong et al. [101], Leonardi et al. [8] —by means of iTRAQ quantitative proteomics analysis of VKC tear samples—found that levels of haemopexin (and also serum albumin and transferrin) were up to 100 times higher than the control tear sample levels; those molecule tear levels also correlated to severity of disease. Additionally, they found that haemopexin, transferrin, mammaglobin B and secretoglobin 1D were significantly overexpressed in VKC tear samples, compared to the control ones [8].

Sacchetti et al. [102] showed that after conjunctival allergen provocation test, tear levels of substance P, CGRP and VIP neuromediators were significantly increased in allergic patients compared to baseline. More recently, in 2015, the group of Leonardi et al. [103] has determined allergen-specific IgE antibodies in tears from active VKC patients and age-matched healthy controls, using a multiplex specific microarray technique for direct measurement of IgE directed against 103 components derived from 47 allergens.

#### Keratoconus

Keratoconus (KC) is the most common degenerative corneal disease [104], whereby structural changes in the cornea cause it to thin and bulge. KC is characterised by a distinctive conical shaping of the cornea and results in diminished vision and reduced quality of life. Despite numerous efforts, no single biomarker for KC has been discovered that allows early diagnosis of the condition. Recently, however, Priyadarsini et al. [105] identified a potentially novel tear fluid marker of KC and termed it gross cystic disease fluid protein-15 (GCDFP-15) or PIP. Another group demonstrated significant changes in the following molecules: RANTES/CCL5, MMP-13, NGF and IL-6 in tear fluids of patients with KC [106]. This group also noted age-dependent associations between IL-13, IL-8/CXCL8, RANTES/CCL5 and MMP-13 and the topographical data. A study by You et al. [107], via ELISA analysis of tear fluids from subjects with KC, showed significantly reduced levels of the glycoprotein secreted frizzled-related protein 1 (SFRP-1). Moreover, tear fluid inflammatory protein expression has been assessed in subjects with KC. Most recently, Shetty et al. [108] noted high levels of MMP-9 and IL-6 in tear fluids of KC patients. This group noted that MMP-9 levels responded (were reduced) to cyclosporine A therapy, thus indicating this protein as a potential target in arresting KC progression. While Sorkhabi et al. [109] identified significantly increased IL-6, IL-1β and IFN-γ levels in KC tears than controls, of note was the fact that this study showed significantly lower levels of the *anti-inflammatory* mediator IL-10 in tears from KC patients.

Other research indicates roles for metabolites related to the urea cycle, TCA cycle and oxidative stress in KC patients, as demonstrated by notable tear fluid changes in proteins associated with these processes [110]. Further evidence for a role in KC of oxidative stress was also shown via lower levels of tear film prolidase activity (PA) in a study of KC patients and healthy subjects [111].

#### Keratopathy

Keratopathy is the term used to refer to any disease or dysfunction of the cornea and can include bullous, band, climatic droplet and neurotrophic keratopathies. Climatic droplet keratopathy (CDK) is a degenerative disease of the cornea, which is characterised by progressive opacity of the cornea’s anterior layers. Proteomics, such as iTRAQ, have been used in numerous studies to define the protein composition of tears from patients with this disorder. For example, Lei et al. [112] used 2D nano-LC-nano-ESI-MS/MS analysis to quantify N-linked glycoproteins in tears from patients with CDK, versus controls. This group found that of the 19 novel N-linked glycoproteins identified in tears, five were found to have significant changes in N-glycosylation levels in CDK patients, compared to normal controls [112]. As N-linked glycoproteins are found in body fluids, they are of particular interest in the field of biomarkers and as potential therapeutic targets. Despite this, very few studies have undertaken tear fluid analysis for N-linked glycoproteins (reviewed in [113]), indicating these proteins may be difficult to assess. Other potential tear fluid biomarkers of CDK include cytokines, MMPs and gelatinases [114, 115]. MMPs have also been indicated in the pathology of another form of keratopathy, diabetic keratopathy. Interest in this particular type of ocular surface disease is on the increase, due to the global phenomenon of rapidly rising rates of diabetes. For example, in a tear study of paediatric patients with type 1 diabetes, researchers reported significantly elevated levels of MMP-9, TIMP-1 and TIMP-2, as well as of MMP-9/TIMP-1 and MMP-9/TIMP-2 ratios versus controls, using ELISA and zymography [116]. Further, they noted a significant correlation between each of MMP-2, MMP-9 and TIMP-2 with Hba1c levels. The authors suggested that the presence of these proteins indicated local tissue remodelling and of local keratopathy disease progression, which may serve as early disease markers. Matsumura et al. [117] investigated the tear fluid levels of MMP-2, MMP-9 and MMP-10 in diabetic patients, pre and post vitrectomy. Using multiplex analysis, they showed significantly higher levels of MMP-10 in the diabetic patients who subsequently developed keratopathy post-surgery, indicating a role for this MMP in mediating post-surgical corneal disorders in diabetes. Tear fluid biomarkers of other diabetes-related ocular disorders, including diabetic retinopathy, will be discussed later.

#### Peripheral ulcerative keratitis

Peripheral ulcerative keratitis (PUK) is a chronic, progressive condition characterised by a crescent-shaped corneal ulcer with epithelial defects adjacent to the limbus [118]. PUK has been linked with various systemic autoimmune conditions, in particular rheumatoid arthritis, Wegener granulomatosis, systemic lupus erythematosus and polychondritis [119, 120]. Tear analysis has been carried out on patients with PUK, investigating the concentrations of MMP-2 and MMP-9. These MMPs have been shown to be elevated in those with PUK [121, 122]. Both of these enzymes are involved in the breakdown of collagen, and in PUK, this relates to the destruction of the cellular structure in the corneal stroma and subsequent corneal perforation. These studies have also shown that the levels of MMP-2 and MMP-9 are increased during active PUK and are reduced during disease inactivity, indicating their involvement in the disease process [121, 122].

#### Trachoma

Trachoma is an infection that is common in developing countries in Africa, the Middle East and Asia and is the most common infectious cause of blindness worldwide. The infectious agent is a bacterium, *Chlamydia trachomatis*, which via trichiasis (ingrowing eyelashes), results in repeated episodes of corneal and conjunctival scarring. An estimated 8 million people are visually impaired as a result of trachoma infection, and a further 84 million suffer from active infection globally (reviewed in [123]). Trachoma is spread by direct contact with eye, nose and throat secretions from affected individuals, or via contact with contaminated clothing. Therefore, as a major cause of preventable blindness worldwide, a reliable test for *C. trachomatis* is necessary in both controlling and eliminating this infection. Numerous programmes have been undertaken, with the aim of reducing both the infection and the clinical signs. There is a long history of detecting immune responses to trachoma via immunoglobulin (Ig) in tears from patients [124–127]. Immune responses have been measured via tear IgG and IgA (against cHSP60, CT795 and CPAF fusion proteins) using ELISA [128], who reported significantly higher IgG antibody levels against cHSP60, CPAF and CT795 in the inflammatory cases of trachoma, versus controls. This group suggested that IgG levels to CPAF may serve as a biomarker for patients at risk of inflammatory trachoma. More recently Mowafy et al. [129] utilised tear ELISA assays to detect IgG and IgM of patients with trachoma.

Finally, in order to improve the understanding of the pathology underlying trachoma infection, Satici et al. [130] examined tear fluid cytokine expression. They found a significant correlation between changes in EGF, TGF-β1 and TNF-α levels and conjunctival scar formation of trachoma patients, indicating a potential role for these inflammatory mediators in scar progression.

### Tear fluid analysis for other ocular disorders

#### Thyroid-associated orbitopathy

Thyroid-associated orbitopathy (TAO) is an autoimmune disease resulting from thyroid dysfunction. TAO is characterised by enlarged extraocular muscles and increased fatty and connective tissue, resulting in protruding eyes, restricted ocular motility and, in severe cases, visual field loss. In recent years, proteomic analysis of tear fluids from patients with TAO has been undertaken in order to better determine disease activity and to stratify patients accordingly. These studies have yielded some interesting data on potential biomarkers of the condition. For example, using CBA, Ujhelyi et al. [131] reported significantly increased levels of tear fluid IL-1β, IL-6, IL-13, IL-17A, IL-18, TNF-α and RANTES/CCL5 in TAO patients versus controls. Of interest, IL-6 was increased 2.5-fold, suggesting this cytokine may serve as a specific marker of disease activity in TAO patients. In another tear cytokine study, Cai et al. [132] reported differing levels of the cytokine IL-7, depending on disease activity states, with the highest IL-7 levels observed in patients with inactive TAO. A later study of patients with active or inactive TAO was performed by Huang et al. [133], and tear fluids were assessed for the cytokines IL-1β, IL-6, IL-7, IL-17A, IFN-γ and TNF-α. They reported that IL-1β was significantly higher in active TAO than inactive disease and controls, while IL-6 and IL-17A were significantly higher in both active and inactive TAO than controls. IL-7 levels were highest overall in inactive TAO among the three groups. Concentration of TNF-α was significantly higher in both active and inactive TAO than for controls [133].

Taken together, these studies indicate a vital role for inflammatory mediators in TAO disease progression and may serve as future diagnostic and/or therapeutic targets for this condition.

#### Aniridia

Aniridia is a rare congenital condition (linked to the PAX6 gene on chromosome 11), whereby the iris is missing or incomplete, and it usually affects both eyes. To date, very few studies have profiled tear fluid proteins in these patients. Recent work by Ihnatko et al. [134] used 2D electrophoresis and liquid chromatography-tandem mass spectrometry (LC-MS/MS) to compare tear proteins in aniridia and control subjects. The authors noted seven differentially expressed proteins in aniridia patients and control subjects, including α-enolase, peroxiredoxin 6, CST4, gelsolin, apolipoprotein A-1, zinc-α2-glycoprotein and lactoferrin. Of these, the former five proteins were more highly expressed in healthy subjects, while the latter two proteins were higher in tears of aniridia patients, and western blot data showed increased tear vascular endothelial growth factor (VEGF) levels in those with aniridia [134]. Further, a recent study by Peral et al. [135] sought to ascertain the tear levels of diadenosine polyphosphates (Ap4A and Ap5A), which have been identified previously as potential dry eye biomarkers, in subjects with aniridia (who also have a propensity to dry eye). In their study of 15 aniridia patients and 40 controls, the authors observed increased levels in aniridia patients (than for controls), which correlated with patient age and corneal disorder progression [135]. Although the research into tear fluid proteomics is still at an early stage in this patient demographic, further biomarker studies are likely to shed light on this condition and may potentially identify therapeutic targets in treating ocular surface complications of this disease.

#### Glaucoma

Glaucoma is an internal ocular condition that usually affects both eyes in which the aqueous humour builds up, causing an increase in intra-ocular pressure. Around 10 % of UK blindness registrations are attributable to glaucoma, and its overall prevalence is approximately around 2 % of people over 40. Moreover, the prevalence of glaucoma is higher in people of black African or black Caribbean descent and those with a family history of glaucoma (reviewed in [136]). Globally, it has been estimated that by 2020, at least 53 million people will be affected by glaucoma [137] and this disease remains the primary cause of irreversible visual impairment.

The main forms of glaucoma include primary open-angle glaucoma (POAG, the most common form), primary angle-closure glaucoma, secondary glaucoma and developmental (or congenital) glaucoma. Due to the devastating effects that untreated glaucoma may have on vision in the working age population, it is imperative that (for improved PPPM) robust biomarkers are identified for drug development and measuring disease progression in this condition [138]. Using ELISA, Ghaffariyeh et al. [139] assessed brain-derived neurotrophic factor (BDNF) in the tear fluids of normal-tension glaucoma (NTG) patients. They found this protein to be substantially reduced in tears of NTG patients versus healthy controls. Pieragostino et al. [140] examined the tears of patients with medically controlled POAG and pseudoexfoliative (secondary) glaucoma, using SDS-PAGE and MALDI-TOF MS. This group demonstrated differing levels of Igs, PIP, lysozyme C, LCN-1 and protein S100, between the two disease subtypes, suggesting different inflammatory pathways underlying the pathologies. This work was followed by the same group observing tear fluid proteomics in treatment-naïve POAG subjects, and they reported up-regulation of 25 proteins in POAG subjects (versus control), 16 of which were inflammatory response mediators [141]. The authors suggested that as a large component of the tear proteins were directly related to inflammatory pathways, these may serve as future biomarkers and/or therapeutic targets of POAG.

In addition, Liu et al. [142] assessed tear fluid MUC5AC by ELISA in 25 POAG patients (versus controls) and reported a reduction following short-term glaucoma medication. By comparison, Roedl et al. [143] found significantly higher levels of tear fluid homocysteine (Hcy, a homologue of the amino acid cysteine) in 36 POAG patients versus controls. This group noted that POAG patients with DED had significantly higher tear fluid levels than POAG patients without DED, indicating that Hcy may serve as a marker for increased risk of both POAG and dry eye in glaucoma patients.

Table 1 summarises the putative biomarkers in tears for ocular diseases.

**Table 1:** Current putative biomarkers in tears for ocular diseases

Disease	Type of molecule	Molecules	References
DED	Proteins	Lysozyme, lactoferrin	Mackie and Seal (1986) [36], Boersma and van Bijsterveld (1987) [37], Goren and Goren (1988) [38]
		LPRR4	Grus et al. (2005) [30], Aluru et al. (2012) [4], Boehm et al. (2013) [33]
		Calgranulin A/S100 A8	Grus et al. (2005) [30], Zhou et al. (2009) [31], Tong et al. (2011) [32], Boehm (2013) [33]
		LPRR3, nasopharyngeal carcinoma-associated PRP 4, α-1 antitrypsin α-enolase, α-1 acid glycoprotein 1	Grus et al. (2005) [30]
		S100 A4, S100 A11 (calgizzarin)	Zhou et al. (2009) [31]
		S100 A9/calgranulin B	Zhou et al. (2009) [31], Tong et al. (2011) [32]
		LCN-1	Tong et al. (2011) [32]
		Mammaglobin B, lipophilin A, B2M	Boehm (2013) [33]
		S100A6, annexin A1 annexin A11, CST4, PLAA	Soria et al. (2013) [34]
		Transferrin, LCN-1	Versura et al. (2013) [35]
		Defensin-1, clusterin, lactotransferrin	Li et al. (2014) [40]
		Cathepsin S	Hamm-Alvarez et al. (2014) [41]
		Anti-SS-A, anti-SS-B, anti-α-fodrin antibodies	Toker et al. (2004) [42], Zandbelt et al. (2009) [43], Yavuz et al. (2006) [44]
		Malate dehydrogenase (MDH) 2	Guo et al. (2014) [21]
		Palate lung nasal clone—PLUNC	Schicht et al. (2015) [46]
	Mucins	(MUC)5AC	Argüeso et al. (2002) [45]
	Neuromediators	NGF, CGRP, NPY	Lambiase et al. (2011) [47]
		Serotonin	Chhadva et al. (2015) [48]
	Cytokines/chemokines	IL-1	Pflugfelder et al. (1999) [51], Solomon et al. (2001) [52], Boehm et al. (2011) [60], Van der Meid et al. (2012) [68], Na et al. (2012) [20]
		IL-2, IL-5	Massingale et al. (2009) [58]
		IL-6	Tishler et al. (1998) [50], Yoon et al. (2007) [53], Lam et al. (2009) [55], Massingale et al. (2009) [58], Boehm et al. (2011) [60], Na et al. (2012) [20], Van der Meid et al. (2012) [68], Lee et al. (2013) [65], Tesón et al. (2013) [70], López-Miguel et al. (2016) [72]
		IL-8/CXCL8	Lam et al. (2009) [55], Massingale et al. (2009) [58], Boehm et al. (2011) [60], Van der Meid et al. (2012) [68], Huang et al. (2012) [61], López-Miguel et al. (2016) [72]
		IL-10	Massingale et al. (2009) [58], Lee et al. (2013) [65]
		IL-12	Lam et al. (2009) [55], Na et al. (2012) [20]
		IL-16, IL-33, GCSF, MCP1/CCL2, MIP1d (CCL15), ENA-78/CXCL5, sILR1, sIL-6R, sgp. sEGFR, sTNFR	Na et al. (2012) [20]
		IL-17A	De Paiva et al. (2009) [57], Na et al. (2012) [20], Lee et al. (2013) [65], Tan et al. (2014) [62]
		IL-21	Lim et al. (2009) [66]
		IL-22	Tan et al. (2014) [62]
		IL-1RA	Enriquez-de-Salamanca et al. (2010) [19], Huang et al. (2012) [61], López-Miguel et al. (2016) [72]
		CXCL9/MIG, CXCL11/I-TAC	Yoon et al. (2010) [59]
		CXCL10/IP-10	Enriquez-de-Salamanca et al. (2010) [19], Yoon et al. (2010) [59]
		MIP-1β/CCL4	Choi et al. (2012) [22]
		RANTES/CCL5	Lam et al. (2009) [55], Choi et al. (2012) [22]
		EGF	Pflugfelder et al. (1999) [51], Ohashi et al. (2003) [64], Lam et al. (2009) [55], Enriquez-de-Salamanca et al. (2010) [19], Tesón et al. (2013) [70], López-Miguel et al. (2016) [72]
		TNF-α	Yoon et al. (2007) [53], Lam et al. (2009) [55], Massingale et al. (2009) [58], Boehm et al. (2011) [60], Van der Meid et al. (2012) [68], Lee et al. (2013) [65]
		INF-γ	Massingale et al. (2009) [58], Boehm et al. (2011) [60], Lee et al. (2013) [65]
		MMP-9	Acera et al. (2008) [54], Chotikavanich et al. (2009) [56], Van der Meid et al. (2012) [68], Aragona et al. (2015) [63], Tesón et al. (2013) [70], López-Miguel et al. (2014) [71], López-Miguel et al. (2016) [72]
		MIP1-α/CCL3	Lam et al. (2009) [55], Choi et al. (2012) [22]
		VEGF	Enriquez-de-Salamanca et al. (2010) [19]
		Fractalkine	Enriquez-de-Salamanca et al. (2010) [19], Na et al. (2012) [20]
	Lipids	OAHFA, lysophospholipids, PUFA-containing diacylglyceride species	Lam et al. (2011) [74], Lam et al. (2014) [75]
		HEL	Wakamatsu et al. (2013) [76]
		HNE, MDA	Choi et al. (2016) [77]
	Metabolites	Cholesterol, *N*-acetylglucosamine, glutamate, creatine, amino-n-butyrate, choline, acetylcholine, arginine, phosphoethanolamine, glucose, phenylalanine	Galbis-Estrada et al. (2014) [77]
Ocular GVHD	Cytokines/chemokines	IL-6, INF-γ	Riemens et al. (2012) [82]
		Soluble TNF receptor 1 (sTNFR1), IL-2, IL-10, IL-17A, TNF-α	Sakimoto et al. (2014) [83], Jung et al. (2015) [84]
		EGF, IL-1RA, IL-8/CXCL8, IP10/CXCL10	Cocho et al. (2016) [85]
Ocular allergy^a^	Cytokines/ chemokines	IL-1α, IL-1β	Leonardi et al. (2006) [94], Sack et al. (2007) [97]
		IL-2	Nivenius et al. (2004) [93], Leonardi et al. (2006) [94], Sack et al. (2007) [97]
		IL-6, IL-12, IL-13, eotaxin-1/CCL11, RANTES/CCL5, MCP-1/CCL2	Leonardi et al. (2006) [94]
		IL-4	Uchio et al. (2000) [88], Nivenius et al. (2004) [93], Leonardi et al. (2006) [94], Sack et al. (2007) [97]
		IL-5	Uchio et al. (2000) [88], Nivenius et al. (2004) [93], Leonardi et al. (2006) [94], Sack et al. (2007) [97]
		IL-10	Nivenius et al. (2004) [93], Leonardi et al. (2006) [94]
		sIL-6R	Shoji et al. (2006) [95], Shoji et al. (2006) [96]
		Eotaxin-2/CCL24	Shoji et al. (2006) [95], Leonardi et al. (2003) [90]
		TNF-α, IFN-γ	Nivenius et al. (2004) [93], Leonardi et al. (2006) [94], Sack et al. (2007) [97]
		TNF-α/IFN-γ, IL-5/IFN-γ, IL-5/IL-10	Cook et al. (2001) [87]
	Other proteins	Neutrophil myeloperoxidase, ECP, eosinophil neurotoxin, sIL-2 receptor, histamine	Leonardi et al. (2003) [89], Abelson et al. (1995) [98], Montan et al. (1996) [99]
		MMP-1 MMP-9 TIMP-2	Leonardi et al. (2003) [91] Leonardi et al. (2003) [91], Acera et al. (2008) [54] Shoji et al. (2006) [95]
		Haemopexin, Substance P, CGRP, VIP Transferrin, mammaglobin B, secretoglobin 1D IgE abs	Pong et al. (2010) [100], Pong et al. (2011) [101], Leonardi et al. (2014) [8] Sacchetti et al. (2011) [102] Leonardi et al. (2014) [8] Leonardi et al. (2015) [103]
KC		GCDFP-15/PIP RANTES/CCL5, MMP-13, NGF, IL-6 MMP-9, IL-6 IL-6, IL-1β and IFN-γ SFRP-1 Prolidase	Priyadarsini et al. (2014) [105] Kolozsvári et al. (2014) [106] Shetty et al. (2015) [108] Sorkhabi et al. (2015) [109] You et al. (2013) [107] Göncü et al. (2015) [111]
Keratopathy		N-linked glycoproteins, cytokines, gelatinases and MMP-2, -9, -10 and TIMP-2	Lei et al. (2009) [112], Holopainen et al. (2011) [114], Holopainen et al. (2012) [115], Symeonidis et al. (2013) [116], Matsumura et al. (2015) [117]
PUK		MMP-2, MMP-9	Geerling et al. (1999) [121], Smith et al. (1999) [122]
Trachoma		Immunoglobulins, IgG against cHSP60, CPAF and CT795 EGF, TGF-β1 and TNF-α	Nema et al. (1977) [124], Sen et al. (1977) [125], Darougar et al. (1978) [126], Mahmoud et al. (1994) [127], Skwor et al. (2010) [128], Mowafy et al. (2014) [129] Satici et al. (2003) [130]
TAO		IL-1β, IL-6, IL-13, IL-17A, IL-18, TNF-α, RANTES/CCL5 IL-7	Ujhelyi et al. (2012) [131], Huang et al. (2014) [133] Cai et al. (2013) [132]
Aniridia		Zinc-α2-glycoprotein, lactoferrin, VEGF Ap4A and Ap5A	Ihnatko et al. (2013) [134] Peral et al. (2015) [135]
Glaucoma		BDNF Immunoglobulins, PIP, lysozyme C, LCN-1, protein S100, lactotransferrin, PRP4, PIP, zinc-alpha-2-glycoprotein, polymeric immunoglobulin receptor, cystatin S, Ig kappa chain C region, Ig alpha-2 chain C region, immunoglobulin J chain, Ig alpha-1 chain MUC5AC Hcy	Ghaffariyeh et al. (2009) [139] Pieragostino et al. (2012) [140], Pieragostino et al. (2013) [141] Liu et al. (2010) [142] Roedl et al. (2010) [143]

^a^The molecules included in this section are those described, regardless of the type of allergy (VKC, AKC, AC, SAC and GPC)

## Tear fluid analysis in systemic diseases

Until recently, little research had been performed on investigating tear fluids for potential biomarkers of systemic diseases, some of which present with ocular surface complications, e.g. diabetes, cystic fibrosis and systemic sclerosis. However, with the improved sensitivity in proteomic detection of low-abundance proteins and the increased interest in retrieval of tissue samples non-invasively for enhanced disease detection, this is likely to change rapidly [23, 25].

### Systemic diseases with ocular complications

#### Diabetic retinopathy

Diabetes mellitus (DM) is a complex metabolic disease, affecting approximately 347 million people globally [144]. DM is known to cause severe retinal disease, such as diabetic retinopathy (DR), which is one of the leading causes of blindness in the working age population. For example, approximately 5 % of the 37 million people suffering from blindness are due to DR. Thus, the predicted global epidemic of DM is likely to result in increasing numbers of patients with associated retinal disease.

Early diagnosis and intervention is crucial in slowing the progression of DR, hence the urgent need for biomarkers to better determine the clinical course. To date, in the UK the presence of DR is determined by clinical evaluation, using retinal screening (reviewed in [145]). The UK is a world leader in diabetic retinal screening, and this programme is highly effective in recognising DR for subsequent monitoring and therapy [146]. Currently, however, no diagnostic test exists for identifying early presentation of DR disease. A non-invasive method of diagnosing early-stage DR would be invaluable in terms of patient treatment and prevention of further retinal and microvasculature damage, ideally prior to clinical presentation.

To date, most studies of DR biomarkers have so far concentrated on assessing aqueous humour markers, including inflammatory cytokines and chemokines [147–149], while others have examined the vitreous [150], or the plasma [151]. Although useful, these studies mainly involve invasive tissue sampling procedures of internal ocular fluids.

In recent years, however, tear fluids have been investigated for the presence of markers for DR. For example, Park et al. [152] investigated tear fluid (and serum) levels of NGF in patients with DR (via ELISA) and noted significantly higher levels in both fluids, versus non-diabetic controls. Furthermore, they reported that levels of NGF correlated with blood glucose levels, duration of DM, HbA1c and diabetic nephropathy, thus indicating NGF’s potential utility as a biomarker. In 2012, Csősz et al. [153] identified six potential candidate biomarkers, including LCN-1, lactotransferrin, lysozyme C, lacritin, lipophilin A and immunoglobulin lambda chain (via nano-HPLC-coupled ESI-MS/MS mass spectrometry) in tears from patients with DR. In the same year, using a combined proteomic analysis approach, Kim et al. [154] identified a number of proteins that were differentially expressed between diabetic groups (with and without DR), versus healthy subjects. Of these proteins, LCN-1, heat shock protein 27 (HSP 27) and B2M were shown to be significantly altered (up- and down-regulated) in the two DM groups, compared with healthy controls. The changes observed in LCN-1, HSP27 and B2M for diabetic patients (with and without evidence of DR) may thus serve as future early diagnostic tools (and/or therapeutic targets) for early-stage DR. While more recently, a tear fluid study by Costagliola et al. [155] reported significantly increased levels of the cytokine tumour necrosis factor alpha (TNF-α) in diabetic patients with DR. Moreover, TNF-α levels were strongly associated with severity of DR. Another recent study by Torok et al. [156] undertook proteomic analysis of tear fluids (via nano-HPLC-coupled ESI-MS/MS mass spectrometry) from 52 diabetic patients, of whom 39 had DR. By combining separate but complimentary techniques (proteomic data with retinal imaging of microaneurysms), this group sought to increase both the sensitivity and the specificity values of photographic screening methods. The ultimate goal is the creation of a less expensive, more user-friendly and more accessible method for clinical DR screening. Although the study showed that the system is not yet fully optimised, they concluded that ongoing developments in this area suggest its future potential in DR screening.

More recently, research by Nguyen-Khuong et al. [157] sought to compare the glycomic profiles of tears from diabetic patients (with and without DR, *n* = 5 each group), versus healthy subjects (*n* = 5), using liquid chromatography (LC)-ESI-IT-MS and tandem mass spectrometry. The authors reported marked conservation of the glycan structures in tear proteins, as well as changes in the glycomic N-linked profile, the latter of which coincided with the onset of diabetes and DR onset. However, this was restricted to the relatively low-abundance (<5 %) glycans. Five low-abundance N-glycans and one O-glycan were significantly altered in patients with DM or DR, versus healthy controls. It should be noted that the authors could not confirm if these changes were linked to specific proteins. This group suggested that marked conservation of the glycan structures of tear proteins between individuals and disease-related changes in low abundance N-glycans may be useful in future tear fluid-based diagnosis of DM progression [157].

#### Systemic sclerosis

Systemic sclerosis (SyS, or scleroderma) is a rare connective tissue disease which is characterised by progressive sclerosis of soft tissues, e.g. thickening of the skin via collagen accumulation, and injuries to small arteries (vasculitis). In severe cases, this will present as vasculitic lesions of the skin, joints, intestines and other major organs, nutrient malabsorption, arthritis, scleroderma and kidney dysfunction. In the eye, SyS commonly presents as dry eye, due to fibrosis of the lacrimal gland, as well as conjunctival telangiectasia and filamentous keratitis [158]. Despite these ocular manifestations, only two studies have investigated tear fluid proteomics in SyS to date [159, 160]. In the first instance, Rentka et al. [159] assessed tear fluid levels of the pro-angiogenic protein, VEGF, in 43 SyS patients and 27 healthy controls. This study demonstrated a reduced average VEGF expression in SyS patients (4.9 pg/L) versus healthy controls (6.15 pg/L), which was not significant. Moreover, the authors concluded that this difference in VEGF levels (20 %) could be due to the decreased tear secretion of SyS patients, indicating that currently VEGF may not serve as a biomarker of the disorder. A further study by this group used proteomics to assessing levels of cytokines via multiplex arrays and CBA [160]. Of the 102 tear fluid cytokines analysed by cytokine array, 9 cytokines were reported to be significantly increased in SyS patients. These were complement factor D (CFD), chitinase-3-like protein 1 (CHI3L1), C-reactive protein (CRP), EGF, interferon-c-inducible protein-10 (IP-10 or CXCL-10), MCP-1, MIG, MMP-9 and vitamin D-binding protein (VDBP). Following this, the sensitive technique of multiplex arrays was used to assay 4 molecules in tears of the 9 SyS patients and 12 controls. The authors demonstrated significantly increased levels of CRP, IP-10 and MCP-1 in tears from SyS patients, versus controls. While CFD tear levels were reduced in SyS patients, these were not found to be statistically significant [160]. This data suggests a role for these specific cytokines in SyS, which may serve both as therapeutic targets and aiding clinical decision-making when recommending artificial tear formulations. It may be argued that as tear fluids represent the local milieu of the eye’s surface, they are a better source of tissue for investigating ocular surface pathologies.

#### Cystic fibrosis

Cystic fibrosis (CF) is a serious genetic disease affecting the exocrine glands, which is characterised by abnormal secretions, leading to mucus build in the lungs, pancreas and intestine, as well as the sweat glands, and affects male fertility. As one of the most common chronic lung diseases in children and young adults, mucus accumulation in CF causes serious lung infections and digestion problems and is a life-threatening disorder. As CF affects the secretory epithelial cells, dry eye is one of the ocular manifestations of this disease. Although the number of tear fluid proteomic analyses has thus far been limited, several articles have investigated the role of cytokines in ocular surface dysfunction of CF patients. For example, the first study to assay tear fluid cytokines was by Mrugacz et al. [161]. This group performed ELISAs to determine IL-8 and IFN-γ levels in tears of patients with CF, alongside ocular surface health assessments. The authors reported significantly higher levels of both cytokines in this patient demographic, versus controls. Interestingly, IL-8 and IFN-γ levels correlated significantly with the clinical severity of CF [161], indicating important roles for these cytokines in both progression of ocular surface inflammation and CF pathology in patients. Thus, IL-8 and IFN-γ may be putative biomarkers for determining dry eye and CF clinical status. Subsequent ELISA studies by this group have reported significantly increased tear fluid levels of the chemokines MIP-1α [162] and MIP-1β [163] in patients with CF, versus healthy subjects. Moreover, in the former study [162] they noted a negative correlation between the CF clinical severity and tear MIP-1α levels, as well as a positive correlation between the MIP-1α and dry eye in CF patients. Their most recent research [163] showed significantly elevated MIP-1β levels in CF patients, of whom those with dry eye syndrome demonstrated significantly raised MIP-1β levels than for CF patients without dry eye. Taken together, these studies indicate that, similarly to tear fluid chemokine/cytokine expression studies in dry eye, these two groups of inflammatory mediators have a role in ocular surface inflammatory response and progression in CF.

### Tear fluid analysis of systemic diseases without ocular complications

#### Cancer

Various articles have reported on the potential of PPPM in addressing the major disease such as cancer and their potential for improved treatment through early detection and more targeted therapies [164, 165].

In the last decade alone, several articles have assessed tear fluids from cancer patients, with a view to finding a non-invasive method of determining markers for this disease. For example, one of the earliest studies was by Evans et al. [166] who investigated tear fluid levels of lacryglobin in breast cancer patients. Lacryglobin is a protein with high homology to mammaglobin A, one of a group of proteins secreted by the lacrimal and salivary glands, uterus and breast that are increased in patients with breast cancer [167]. In their novel study, Evans et al. [166] used 1D and 2D electrophoresis of tear samples from patients with various cancers, in order to determine the usefulness of tear fluid screening. They reported that tear fluid lacryglobin was present primarily in patients with colon (100 %) or prostate cancer (100 %), followed by cancers of the breast (88 %), lung (83 %) and ovary (33 %). Three (60 %) of the control subjects showed lacryglobin presence, and, of note, two of these subjects had a family history of breast and prostate cancer [166]. This early report thus indicated the potential of tear fluid lacryglobin as a non-invasive biomarker of cancer.

As the second most common cause of cancer mortality in women, breast cancer has been of particular interest with regard to identifying early diagnostic markers, as early diagnosis is crucial in reducing mortality [168]. Despite the existence of serum markers such as CEA, CA15-3 and CA27.29 for monitoring metastatic disease [169], no definitive biomarkers are available for the early detection of breast cancer in patients with smaller lesions. Therefore, two studies [170, 171] of tear fluid proteins from patients with breast cancer were undertaken in order to identify novel early-stage biomarkers of the disease. Using surface-enhanced laser desorption/ionisation-time-of-flight mass spectroscopy (SELDI-TOF-MS), they reported significant differences in tear (and blood) proteins between breast cancer patients and healthy, age-matched controls, showing 90 % specificity and sensitivity [170]. This group’s follow-up SELDI-TOF-MS study of tear fluid protein profiles from 50 breast cancer patients demonstrated significant differences in a panel of 20 biomarkers (versus healthy controls), with an overall specificity and sensitivity of 70 % [171]. Taken together, their data indicates that, as well as being highly accessible and easily retrieved, tear fluid samples are less complex (protein content-wise), thus making them ideal tissue samples for biomarker identification of breast cancer, via SELDI-TOF-MS. Finally, Böhm et al. [172] used MALDI-TOF-TOF analysis of tears from breast cancer patients and showed a distinctive difference in 20 biomarkers, versus healthy controls. As the principal cause of death in women globally [173], it is imperative that robust biomarkers for early detection of breast cancer are identified, ideally in an easily accessible body fluid. The above studies indicate the usefulness of tear fluid protein profiling in achieving this goal.

#### Neurological disorders

##### Multiple sclerosis

Multiple sclerosis (MS) is the most common neurological disorder of early adult life in the UK [174] and results from chronic demyelination of the central nervous system (CNS). Diagnosis usually involves several tests, including magnetic resonance imaging (MRI), and a final confirmation may take years to achieve. Although published literature in the field has been limited to date, tear analysis was first suggested for the diagnosis of MS as far back as the mid-1980s [175, 176]. Since then, various groups have performed tear studies in order to better determine biomarkers of the disease. As mentioned earlier, several tests are used to confirm an MS diagnosis. The most consistent biomarker of MS to date has been the presence of IgG oligoclonal bands in cerebrospinal fluid (CSF), which was reviewed in 2006 [177]. Yet, retrieval of CSF via lumbar puncture is a highly invasive procedure for patients, involving a non-insignificant degree of discomfort. Hence, there is a necessity for an earlier, and less invasive, diagnosis of this debilitating disorder.

Early work by Coyle et al. [176] was the first study that used isoelectric focusing (IEF) to identify oligoclonal bands of IgG in tears, showing their presence in 67 % of the MS patients tested (14/21). By contrast, however, Mavra et al. [178] performed IEF and reported no IgG bands for patients with either MS or optic neuritis, while 16/20 patients with either infections of the CNS or the presence of systemic immune disorders did present with these bands. Similarly, Martino et al. [179] utilised IEF of tears to identify differential oligoclonal bands, as an alternative to lumbar punctures. Small increases of total IgG, IgM and IgA levels were noted in tears from patients with MS and other neurological disorders, versus controls. Yet only one MS patient showed *unique* bands in tears, which were not observed in the paired CSF and serum. This group thus postulated that the main polyclonal Ig in MS was IgG. Latterly, Devos et al. [180] indicated that the specificity and sensitivity of oligoclonal bands of IgG in tears from MS patients was similar to that of CSF and far less invasive. This group indicated that tear fluid should therefore be investigated as a valuable biological material for biomarker measurements of MS. Taken together, the literature so far on oligoclonal bands in tear fluids appears to be contentious. Not least, following work by Lebrun et al. [181] who recently showed oligoclonal bands in radiologically isolated syndrome (RIS), this newly identified entity defines patients who have white matter lesions, without clinically defined symptoms of MS. Therefore, whether oligoclonal bands are more indicative of this MS-type disorder remains to be seen and warrants further investigation.

Aside from oligoclonal bands, recent proteomic analysis by Salvisberg et al. [182] has investigated other tear fluid protein markers of MS. This group performed three independent quantitative (tandem mass tag) experiments on tears from patients with MS versus healthy controls. Of the 185 tear fluid proteins identified, 42 were differentially expressed. Of these, α-1-antichymotrypsin was the only molecule to be significantly elevated across all three experiments (*p* < 0.05). The authors concluded that raised tear fluid levels of this acute inflammatory protein could serve as a future MS biomarker, which could replace traditional lumbar punctures. Salvisberg’s study is the most recent wholescale proteomic tear fluid analysis in MS, showing much more research is necessary for this particular condition before a definitive biomarker is confirmed.

##### Parkinson’s disease

Parkinson’s disease (PD) is a progressive, degenerative neurological condition of the CNS that predominantly affects the motor system. It is the second most common neurodegenerative disease after Alzheimer’s disease [183] and affects approximately 1.2 million people in Europe. As with other systemic diseases, research into tear fluid biomarkers is at a very early stage. Most PD tear fluid studies have involved assessing the quality and stability of the tear film in these patients [184]. Yet in 2013, a multiplex array study of tear fluids from 18 patients with PD versus 17 healthy controls compared tear TNF-α levels alongside clinical characteristics [185]. This group noted significantly higher levels of TNF-α in patients with PD than for controls (*p* = 0.02), despite these levels not being linked to PD duration or severity. The authors concluded that tear fluid analysis was a suitable methodology for investigating biomarkers and that TNF-α may be a marker of neurological inflammation in PD patients [185].

Following this work, Börger et al. [186] recently proposed the usefulness of tears as a source of PD biomarkers. The authors are currently performing a “monocentric, prospective, diagnostic trial” in which they are retrieving tear fluids from PD patients, as well as from atypical Parkinsonian syndromes, and healthy controls. In addition to completing clinical characterisation, all tissue samples (tear fluid, CSF and blood) will be analysed via LC ESI-MS and the authors hope this prospective study will provide an understanding of proteomic alterations in PD and thus identify novel prospective biomarkers of the condition [186].

Table 2 summarises the putative biomarkers in tears for systemic diseases.

**Table 2:** Putative tear fluid biomarkers of systemic diseases

Disease	Molecules	References
Cancer	Lacryglobin Sulf-1, cystatin SA, 5-AMP-activated protein kinase subunit γ-3, triosephosphate isomerase, microtubule-associated tumour suppressor 1, keratin (type I) putative LCN-1 like protein, malate dehydrogenase, Ig α-2 chain c region, Ig heavy chain VIII region (BRO% WEA), protein S100-A4, keratin (type II), pericentrin, complement C1q subcomponent subunit C	Evans et al. (2001) [166] Böhm et al. (2012) [172]
DR	NGF, LCN-1, lactotransferrin, lysozyme C, lacritin, lipophilin A, Ig lambda chain LCN-1, HSP27, B2M TNF-α N- and O-linked glycans	Park et al. (2008) [152] Csősz et al. (2012) [153] Kim et al. (2012) [154] Costagliola et al. (2015) [155] Nguyen-Khuong et al. (2015) [157]
MS PD	IgG α-Antichymotrypsin TNF-α	Coyle et al. (1987) [176], Martino et al. (1993) [179], Devos et al. (2001) [180] Salvisberg et al. (2014) [182] Çomoğlu et al. (2013) [185]
SyS	CFD, CHI3L1, CRP, EGF, IP-10, MCP-1, MIG, MMP-9, VDBP	Rentka et al. (2015, 2016) [159, 160]
CF	IL-8, IFN-γ MIP-1α, MIP-1β	Mrugacz et al. (2006, 2007, 2010) [161–163]

## Conclusions

It can be seen that the field of tear fluid analysis for earlier diagnosis of ocular and systemic disease is undergoing a sea change. Technological advances, as well as increased scientific and clinical interest in the non-invasive methods of sampling from patients, have all served to make the field of tear fluid analysis an attractive option for disease diagnosis and monitoring. Promising results have been shown in those studies reviewed here (see Tables 1 and 2 for summaries of putative tear fluid biomarkers to date). Further developments in the fields of proteomic, lipidomic and metabolomic detection may serve to improve personalised medicine of patients in the near future. Of particular interest is the recent strategic document, the “EPMA White paper 2012” [187] which discusses the paradigm shift from reactive to PPPM. This change in responses to treating disease is crucial for the development of innovative medical fields such as biomarker research, as well as in the personalised clinical application of therapies. It is anticipated that, ultimately, this change in diagnosis and therapy will help in the future design and development of new, more selective and effective therapies for each individual patient. Standardisation of tear fluid retrieval, processing and storage are, however, necessary before these developments can be fully recognised and implemented in patient care. Also, establishment of the “normal” molecule concentration value range in healthy subjects, taking into consideration both age and gender, will be necessary.

## Abbreviations

AKC, atopic keratoconjunctivitis; CBA, cytometric bead array; CDK, climatic droplet keratopathy; CF, cystic fibrosis; CNS, central nervous system; CSF, cerebrospinal fluid; DED, dry eye disease; DIGE, differential gel electrophoresis; DM, diabetes mellitus; DR, diabetic retinopathy; DRYaq, aqueous-deficient dry eye; DRYlip, lipid-deficient dry eye; FDA, Food and Drug Administration; GPC, giant papillary conjunctivitis; GVHD, graft versus host disease; IEF, isoelectric focusing; IL, interleukin; KC, keratoconus; LCN-1, lipocalin-1; LFU, lacrimal functional unit; MGD, meibomian gland dysfunction; MMP, matrix metalloproteinase; MS, multiple sclerosis; NIH, National Institute of Health; NMR, nuclear magnetic resonance; PD, Parkinson’s disease; POAG: primary open-angle glaucoma; PUK, peripheral ulcerative keratitis; RIS, radiologically isolated syndrome; SAC, seasonal allergic conjunctivitis; SELDI-TOF-MS, surface-enhanced laser desorption/ionisation-time-of-flight mass spectroscopy; SS, Sjögren’s syndrome; SyS, systemic sclerosis; TAO, thyroid-associated orbitopathy; VKC, vernal keratoconjunctivitis
